# Impact of Chlorine Dioxide on Pathogenic Waterborne Microorganisms Occurring in Dental Chair Units

**DOI:** 10.3390/microorganisms11051123

**Published:** 2023-04-26

**Authors:** Theresa Isabella Maria Krüger, Susann Herzog, Alexander Mellmann, Thorsten Kuczius

**Affiliations:** Institute of Hygiene, University Hospital Münster, Robert Koch-Straße 41, 48149 Münster, Germany; t_krue12@uni-muenster.de (T.I.M.K.); susann.herzog@ukmuenster.de (S.H.); alexander.mellmann@ukmuenster.de (A.M.)

**Keywords:** chlorine dioxide, infection control, dental unit waterline, *Legionella*, *Pseudomonas aeruginosa*, disinfection, *Staphylococcus aureus*

## Abstract

Bacterial contamination is a problem in dental unit water lines with the consequence of implementing regular disinfection. In this study, the short-term impact of chlorine dioxide (ClO_2_) treatment was investigated on the microorganisms *Legionella pneumophila* and *L. anisa*, *Pseudomonas aeruginosa*, *Escherichia coli,* and *Staphylococcus aureus*. The environmental background was proven as an important factor regarding the tolerance to 0.4 mg/L ClO_2_ as saline and phosphate-buffered saline resulted in a higher bacterial reduction than tap water. Gram-positive microorganisms demonstrated higher robustness to ClO_2_ than Gram-negative, and microorganisms adapted to tap water showed increased stability compared to cultured cells. At high densities, substantial numbers of bacteria were able to withstand disinfection, whereby the use of 4.6 mg/L ClO_2_ increased the inactivation rate. A massive cell decrease occurred within the first 5 minutes with subsequent plateau formation or slowed cell reduction upon further exposure. This biphasic kinetics cannot be explained by a ClO_2_ depletion effect alone, because the probability of bacterial subpopulations with increased tolerance should be taken into account, too. Our results prove high disinfection efficiency to microorganisms that were rather found in correlation to the level of bacterial contamination and background solutions than the chosen concentration for ClO_2_ treatment itself.

## 1. Introduction

The quality of water in dental unit water lines (DUWL) is of considerable importance for safety and health in dental practices, whereby a high microbiological load of DUWLs is a widespread, long-known, and still current problem [1,2,3]. The main reason for microbiological contamination of DUWLs is generally the presence of microorganisms in tap water [4,5], composed of non-pathogenic microorganisms and potentially opportunistic pathogens. Another risk factor for the contamination of DUWLs is retrograde contamination by aspiration of patients’ saliva [6,7]. Stagnation conditions and biofilm formation promote an increase in cell numbers as well [5,8]. Both dental staff and patients are persistently exposed to water directly or to aerosols due to the regular and repeated use of water-bearing instruments such as ultrasonic scalers and high-speed dental handpieces [9,10]. The regular monitoring for microbiological contamination of dental chair units is recommended by the Robert Koch Institute´s guideline for infection prevention in dentistry [11] with the determination of the total bacterial count and presence of *Legionella* species. The Gram-negative *Legionella* are mainly present in warm water lines [12]. They were detected in 68% of the water samples recovered from DUWLs and 8% were identified as *Legionella pneumophila (L. pneumophila)* [13]. *Legionella anisa (L. anisa)* was found in DUWLs as well [14]. Inhalation or aspiration of contaminated aerosols and water can cause Pontiac fever or legionellosis with life-threatening symptoms [15,16]. Both diseases are predominantly related to water contamination by *L. pneumophila* [17] and *L.* non-*pneumophila*, even after visits to dental practices [18,19].

Another important waterborne microorganism is the opportunistic pathogen *Pseudomonas aeruginosa (P. aeruginosa)* which causes a variety of life-threatening infections such as pneumonia, bacteremia, and wound infection [20,21]. It was shown that 93.3% out of 300 water samples tested from DUWLs were contaminated with *Pseudomonas* species [22]. In 10 out of 44 samples tested from DUWLs contamination with *P. aeruginosa* could be observed [23]. Case studies reported human infections with *P. aeruginosa* in medically pre-diseased patients after dental visits [24]. In addition to these Gram-negative waterborne microorganisms, Gram-positive bacteria are present in water as well. *Staphylococcus aureus (S. aureus)* is an example of human skin commensal [25], which also was found in DUWLs as contamination [26].

Regular disinfection of the DUWLs based on hydrogen peroxide [27] and chlorine in the form of sodium hypochlorite [28] is used to reduce bacterial colonization by 3–5 log_10_ levels or 99.9–99.999% [29]. Chlorine is a powerful agent due to its high oxidizing capacity, however, with the disadvantage of lacking efficacy outside a narrow pH range [30] and formation of hazardous by-products such as trihalomethanes (THMs) and haloacetic acids (HAAs) [31]. An alternative approach is the use of chlorine dioxide (ClO_2_) [32,33], which is valued as a potent, selective oxidizing, and stable agent over a wide pH range [30,34,35]. Moreover, ClO_2_ forms no significant amounts of by-products as THMs and HAAs [36], and its microbicidal effect is proven to be wide-ranged [37,38,39] and comparable with or better than chlorine [30,34]. Compared to sodium hypochlorite and hydrogen peroxide as alternative disinfectants [27,28], chlorine dioxide was found the more potent bactericide [40,41], even though it is more costly [42]. As the sole agent or as a supplement to others, ClO_2_ is already in use for the disinfection of potable water from drinking water treatment plants [43], food products [44], medical equipment [45], and for combating *Legionella* in hospitals water systems [46]. Yet, the exact bactericidal effect of ClO_2_ is still unknown.

In this study, we investigated the short-term impact of ClO_2_ on the disinfection of specific planktonic microorganisms typically contaminating water lines and DUWLs. We focused on the efficiency of microbial inactivation directly in tap water, which is the natural habitat of microorganisms. The tolerance and sensitivity to ClO_2_ treatment were analyzed regarding two physiological conditions of microorganisms: agar cultivation for the depiction of young bacteria and tap water adaption for a prolonged time to mimic real conditions of the water environment.

## 2. Materials and Methods

### 2.1. Bacterial Isolates and Strains

The monitoring of microbiological contamination of dental chair units aims at the detection of *Legionella* with *L. pneumophila* as a high-risk pathogen and *L.* non-*pneumophila* frequently determined in dental chairs. In case of increased bacterial counts, it is recommended by the Robert Koch Institute´s guideline for infection prevention in dentistry [11] to check for the presence of *P. aeruginosa* as an opportunistic waterborne pathogen. The Gram-positive strain S. *aureus* as a classic human skin microorganism and the Gram-negative *Escherichia coli (E. coli)* as a fecal bacterium can be transmitted to dental units via patient contacts. Table 1 summarizes the isolate and strains used in this study. The reference strains, originating from American Type Culture Collection, were obtained from the German Collection of Microorganisms and Cell Cultures (Leibniz Institute DSMZ, Heidelberg, Germany). The environmental isolate of *L. anisa* was received from a water sample of a dental chair by routine analyses according to recommendations of the Robert Koch Institute Germany [11]. *L. anisa* was identified by cultivation on buffered charcoal yeast extract with glycine, vancomycin, polymyxin B, cycloheximide (GVPC) agar (Xebios, Düsseldorf, Germany), and the species was determined using the biotyping technique (Bruker MALDI Biotyper, Bremen, Germany).

### 2.2. Adaption of Microorganisms to Tap Water and Survival Duration

As microorganisms may survive in water for a long time accompanied by physiological changes, we investigated the stability of this distinctive water-adapted status to ClO_2_ treatment. To obtain an intensive cell density, single colonies from a Luria lysogeny broth (LB) agar plate (Roth, Karlsruhe, Germany) were suspended in an LB culture medium (Roth, Karlsruhe, Germany) following incubation at 36 °C overnight under constant rotation at 180 rpm. For the successive adaption to tap water, the sterilized institute´s tap water was used with the physical parameters of pH 8.4, the conductivity of 490 µS/cm, and oxidizability of 1.6 O_2_/mL, measured according to DIN EN ISO 10523, DIN EN 27888 and DIN EN ISO 8467, respectively. The overnight culture was mixed with the doubled volume of sterile tap water and incubation continued at 25 °C under low shaking conditions (96 rpm) for 4 days. Finally, microorganisms were harvested by centrifugation (2000× *g* for 20 min) and the pellet was resuspended in sterile tap water in half of the initial volume. Incubation continued for the experimental test period at 25 °C with constant low rotation (96 rpm) to avoid biofilm formation. 

Colonies of *L. pneumophila* serogroup 1 (ATCC 33152) and *L. anisa* (DSM 17627), were grown on buffered charcoal yeast extract agar (BCYE) supplemented with cysteine (Xebios, Düsseldorf, Germany), were inoculated directly in sterile tap water resulting in a density of 9–10 log_10_ cells/mL. Incubation followed at 25 °C under low shaking (96 rpm) for a prolonged time period.

Aliquots of tap water-adapted bacteria were periodically analyzed for cultivability by dropping the suspensions in tenfold dilutions in a volume of 10 µL as triplicates on LB agar or in the case of *Legionella* on GVPC agar plates. Following incubation at 36 °C, plates were inspected until growth was visible, and resulting colony-forming units (CFUs) were counted.

### 2.3. ClO_2_ and Inactivation Solutions

The ClO_2_ solution (0.6% (*v*/*v*); Clorious2; Brenntag, Essen, Germany) was stored at 4 °C in the dark and was pre-diluted freshly in filtered deionized water before use in each case. The appropriate volume ratios were used in the experiments so that the final ClO_2_ concentrations resulted in 0.4 mg/L, 1.0 mg/L, and 4.6 mg/L. Samples were incubated for the time intervals of 1 min, 2.5 min, 5 min, 15 min, and 30 min together with the indicated ClO_2_ concentrations. After time intervals, the ClO_2_ reactions were stopped by the addition of sodium thiosulfate (Na_2_S_2_O_3_) (Merck, Darmstadt, Germany) ranging in final concentrations about sevenfold excess of ClO_2_ in the respective assays. 

### 2.4. ClO_2_ Treatment of Microorganisms and Re-Cultivation on Solid Media

The microorganisms were inoculated onto LB agar and on BCYE agar plates with cysteine followed by incubation at 36 °C for approximately 18 h for non-*Legionella* and 72 to 96 h for *Legionella* species, respectively. Well-grown colonies were serially suspended in either sterilized tap water (Institute for Hygiene, Münster, Germany), saline (0.85% (*w*/*v*) NaCl; Merck, Darmstadt, Germany), or phosphate-buffered saline (PBS) (30 mM Na_2_HPO_4_/KH_2_PO_4_; pH 7.2) and each adjusted to a cell density of approximately 10^7^ to 10^8^ cfu/mL. As *S. aureus* tended to aggregate, cells were sonicated carefully in an ultrasonic bath (Bandelin, Berlin, Germany) for 5 min and 35 kHz. Cell suspensions were diluted serially tenfold in the respective suspension medium to 10^4^ cfu/mL. The microorganisms, which were adapted to tap water, were diluted tenfold in tap water in turn. The ClO_2_ aliquots were added in concentrations as indicated to each bacterial dilution. The reactions were left to act for indicated time intervals at room temperature prior to inactivation using the Na_2_S_2_O_3_ stop solution. 

For cell counting, samples were diluted again in steps of ten, and volumes of 10 µL were spotted as triplicates on agar plates, non-*Legionella* on R2A, and *Legionella* species on GVPC. The CFUs were counted finally after 48 h at 36 °C for non-*Legionella* and after 10 days for *Legionella* species.

### 2.5. Effect of ClO_2_ Depletion

To investigate a depletion effect under our conditions, two concentrations (10^3^ and 10^6^ cfu/mL) of heat-killed microorganisms (90 °C for 5 min) of *E. coli* and *P. aeruginosa* were mixed with ClO_2_ at a final concentration of 0.9 mg/L. After 5 min pre-incubation, living cells of the respective species were added at a final concentration of 10^6^ cfu/mL. Reactions were stopped with the application of Na_2_S_2_O_3_ after a further 5 min incubation. Survival was analyzed by CFU determination as described above.

### 2.6. Analysis of the Data

Each series of tests was carried out at least two to three times. The plots given here refer to the minimum effect of ClO_2_ in a test batch. 

The cell counts of each dilution were determined in triplicates. The total number was determined from the countable dilutions as arithmetic means (±standard deviation). 

Regarding the ClO_2_ depletion experiment, analyses with *E. coli* and *P. aeruginosa* were performed twice each. In respective of the analyzed bacteria species, the arithmetic means for both test runs were calculated and finally, single approaches were statistically compared by the one-way analysis of variance (ANOVA) test using the Bonferroni post-test.

## 3. Results

### 3.1. Impact of the Suspension Background for Bacterial Inactivation by ClO_2_

**Figure 1:**
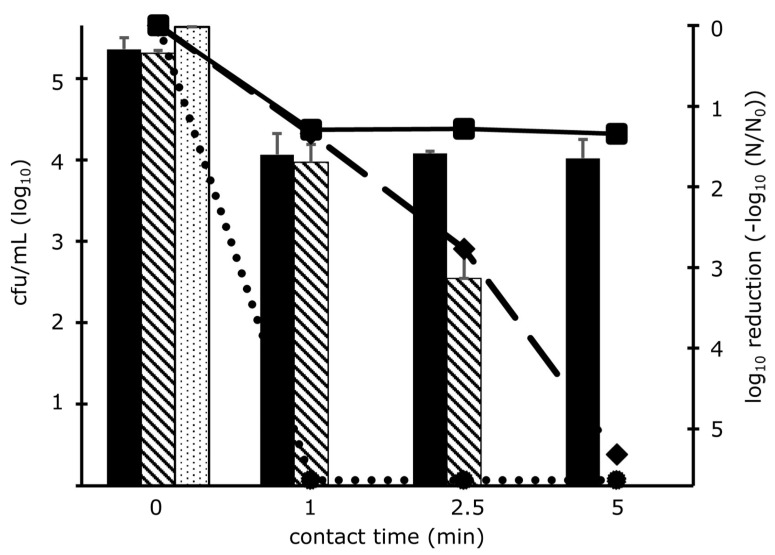
Impact of the medium of suspended microorganisms on ClO_2_ treatment: *E. coli* cells, given as cfu/mL on a log_10_ scale, were suspended in tap water (black columns), physiological saline (striped columns) and PBS (dotted columns) followed by treatment with 0.4 mg/L ClO_2_ for several minutes as indicated. Additionally, the course of short-time inactivation in the respective suspension media is displayed as log_10_-reduction for tap water (solid line), saline (dashed line), and PBS (dotted line). The mean values are presented from one experiment; the cell counts of each dilution were determined in triplicates. The total number was determined from the countable dilutions as arithmetic means (±standard deviation).

The environmental background in which microorganisms are present may have an impact on cell stability and tolerance to ClO_2_. Therefore, *E. coli*, a well-characterized and fast-growing bacterium, was suspended in different media to investigate the biological stability on the one hand and the efficiency of the disinfection agent on the other hand. Based on a bacterial concentration of 10^5^ cfu/mL and exposure to 0.4 mg/L ClO_2_, complete inactivation of all cultivable microorganisms was achieved, when *E. coli* was taken up in PBS (Figure 1). In contrast, a successive decrease of living bacteria followed over time when suspended in physiologic saline. Counts dropped down gradually by 1.3 and 2.8 log_10_-levels within the first and after 2.5 min, respectively. After 5 min of ClO_2_ incubation in saline, no growth could be observed. However, microorganisms solved in tap water showed high stability. In the first minute of incubation, the cell number demonstrated a 1.3 log_10_-reduction and remained stable during further incubation.

For the conduct of experiments, bacteria were suspended in tap water, because it is the natural environmental background for microorganisms in pipes and dental chair units.

### 3.2. Adaption of Microorganisms to Tap Water and Survival Monitoring

**Figure 2:**
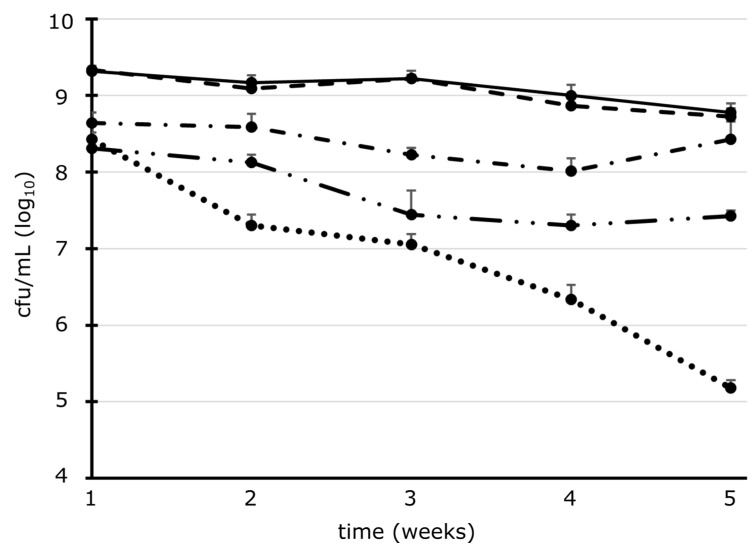
Survival of microorganisms in tap water over time: The survival rates of the reference strains *P. aeruginosa* (solid line), *E. coli* (dashed line), *L. anisa* (one dot-dashed line), *L. pneumophila* serogroup 1 (double dot-dashed line) and *S. aureus* (dotted line), given as cfu/mL, were followed over time after adapting to tap water. The survival curves over a period of five weeks are shown. During this time interval, the cell count decreased by less than one log_10_ stage for *P. aeruginosa*, *E. coli,* and the *Legionella* species. The survival rate of *S. aureus* decreased continuously over time. Plate counting was carried out in triplicates (±standard deviation) at time points as indicated.

When microorganisms colonize as planktonic bacteria in the water supply system for a longer time period, their physiology may change as a reaction to stress conditions and starvation states. To simulate this condition, we adapted the microorganisms to tap water and monitored their survival over an extended time period using cultivation (Figure 2). During the five weeks incubation period, the survival rate decreased by less than one log_10_-level for all Gram-negative strains *P. aeruginosa*, *E. coli,* and the *Legionella* species. The survival curve for *S. aureus* decreased clearly over time.

### 3.3. Kinetics of the Disinfection Efficacy of ClO_2_ on Waterborne Microorganisms

**Figure 3:**
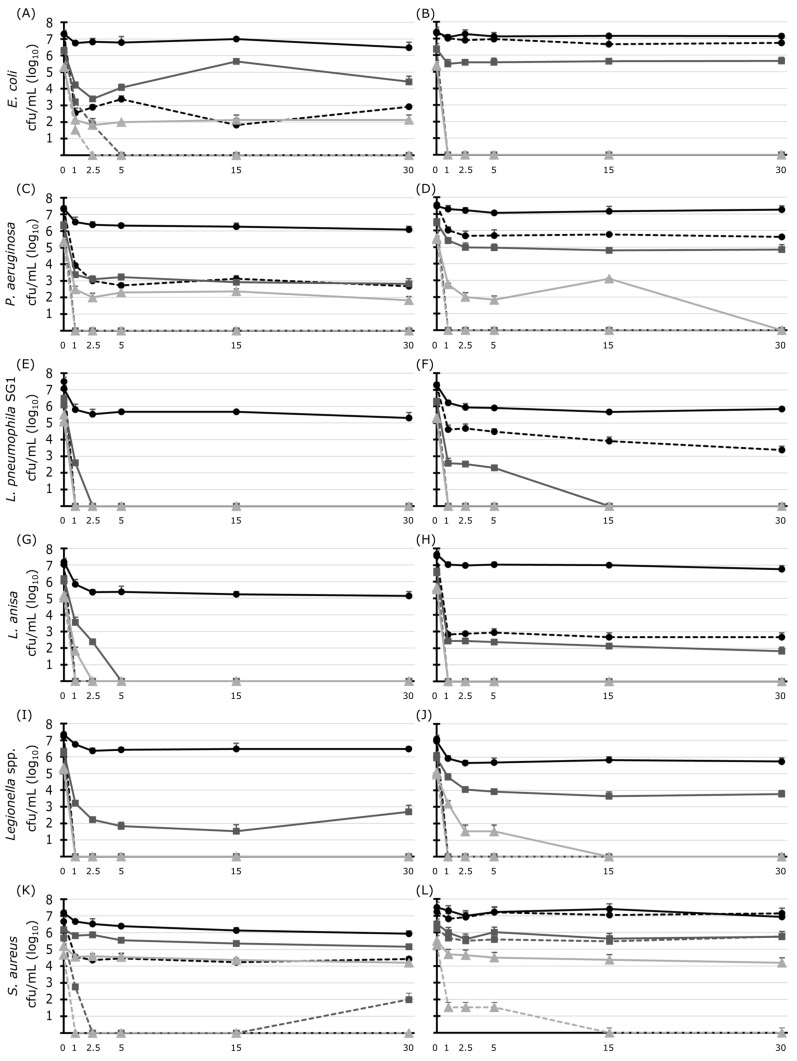
Inactivation kinetics of microorganisms relevant to dental chair units after ClO_2_ exposure over time: Several microbial species were subjected to a treatment with 1.0 mg/L (solid line) and 4.6 mg/L (dashed line) ClO_2_ and were incubated for the time intervals of 1 min, 2.5 min, 5 min, 15 min, and 30 min. Bacteria were taken directly from cultivation plates (**A**,**C**,**E**,**G**,**I**–**K**), as well as after adaption to tap water (**B**,**D**,**F**,**H**,**L**): the reference strains *E. coli* (**A**,**B**), *P. aeruginosa* (**C**,**D**), *L. pneumophila* serogroup 1 (**E**,**F**), *L. anisa* (**G**,**H**), the *Legionella* species (spp.) with *L. pneumophila* serogroup 5 (**I**) and the isolate *L. anisa* (**J**), and the reference strain *S. aureus* (**K**,**L**). The inactivation kinetics were determined for the cell concentrations of 10^7^ (black lines with circles), 10^6^ (dark grey lines with squares), and 10^5^ (grey lines with triangles). Plate counting, given as cfu/mL, was carried out in triplicates (±standard deviation) at time points as indicated; values represent the mean values from the minimum effect of ClO_2_ in a test series.

The inactivation kinetics of several bacterial species, taken directly from agar plates or after adaption to tap water, were analyzed over time when suspended in tap water and treated with 1.0 and 4.6 mg/L ClO_2_ (Figure 3). Higher concentrations of the disinfectant caused more intense inactivation of all microorganisms. The impact over time played a lesser role (Figure 3). 

*E. coli* (Figure 3A,B) and *P. aeruginosa* (Figure 3C,D)*,* both cultivated on plates overnight and adapted to tap water, respectively, with an initial cell density of 10^7^ cfu/mL and treated with 1 mg/L ClO_2_, showed a low decrease within the first minutes following plateau formation (maximum decrease of 1.3 log_10_-levels). When adjusted to 10^6^ cfu/mL, bacterial counts were reduced by maximal 3.5 log_10_-levels for the overnight culture and maximal 1.6 log_10_-levels for the water-adapted culture after 30 min. At the lower density of 10^5^ cfu/mL and after 30 min, the counts were reduced by maximal 3.5 log_10_-levels for the overnight culture. In contrast, tap water-adapted strains were eradicated after this time. When ClO_2_ was added with a high concentration of 4.6 mg/L, a maximum decrease of 5.5 log_10_-levels was achieved for a cell density of 10^7^ cfu/mL with overnight plate-cultured cells. In contrast, tap water-adapted cells proved high tolerance to this treatment with a maximal decline of 1.9 log_10_-levels. At lower densities of 10^6^ and 10^5^ cfu/mL and after 5 and 2.5 min contact time, agar-cultivated *E. coli* failed to grow entirely, whereas *P. aeruginosa* showed no growth after 1 min. Tap water-adapted cells failed to grow under these conditions.

**Figure 4:**
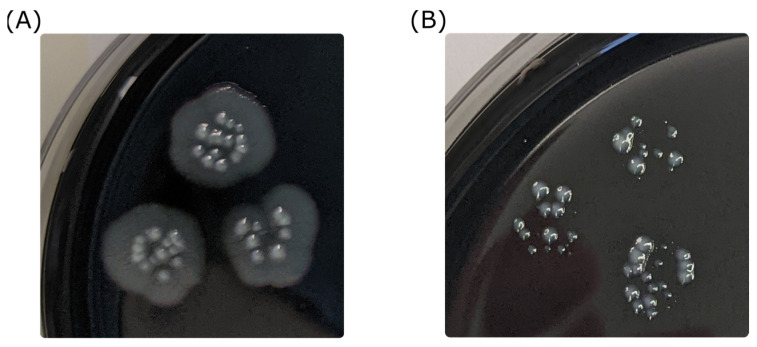
Differential colony morphology of *L. pneumophila* after ClO_2_ treatment: *L. pneumophila* serogroup 5 colonies that survived a 1 mg/L ClO_2_ treatment after 1 min showed a variable morphology with delayed growth (**B**) compared with untreated colonies (**A**) after 5 days of incubation. The two image sections are on the same scale.

Both serogroup representatives of *L. pneumophila* at 10^7^ cfu/mL from different cultivations demonstrated a low reduction of cell numbers with plateau formation when treated with 1 mg/L ClO_2_ (Figure 3E,F,I). With an initial cell density of 10^6^ cfu/mL, agar-cultivated *L. pneumophila* serogroup 1 was eradicated after 2.5 min but the tap water-adapted showed elimination only after 15 min of incubation. *L. pneumophila* serogroup 5 achieved disinfection standard with >3 log_10_-levels cell reduction. Using 4.6 mg/L ClO_2_, only tap water-adapted *L. pneumophila* serogroup 1 at 10^7^ cfu/mL was able to survive. Interestingly, we could detect different growth behavior patterns with rapid and delayed growth and atypical morphology (Figure 4). 

Using 10^7^ cfu/mL, the inactivation kinetics of the *L. anisa* microorganisms showed plateau formation and cell reduction among 0.6 and 2.0 log_10_-levels (Figure 3G,H,J) after treatment with 1 mg/L ClO_2_. Lower densities of *L. anisa* from the plate failed in growth starting after 5 min and 2.5 min of incubation. On the other hand, *L. anisa* both tap water-adapted and as an environmental isolate showed growth and plateau formation at an initial cell density of 10^6^ cfu/mL during the treatment with 1 mg/L ClO_2_. Growth failed at lower cell densities after 1 min for the tap water-adapted strain and after 15 min for the dental chair isolate. Treated with 4.6 mg/L ClO_2_, only the tap water-adapted *L. anisa* at a cell density of 10^7^ cfu/mL was able to survive and demonstrated growth in association with a decrease of >4 log_10_-levels after 1 min.

The Gram-positive *S. aureus* demonstrated marking higher stability to ClO_2_-treatment compared with the Gram-negative microorganisms (Figure 3K,L). Regardless of the cultivation manner, treatment with 1 mg/L ClO_2_ caused a reduction of *S. aureus* by 0.1 and 1.3 log_10_-levels only that were independent of incubation time and the initial cell density. Similar to other findings in this work, *S. aureus* in high density demonstrated plateau formation when treated with 4.6 mg/L ClO_2_, whereas disinfection or elimination occurred only with a low initial cell density.

### 3.4. Analysis for ClO_2_ Depletion on Selected Microorganisms

**Figure 5:**
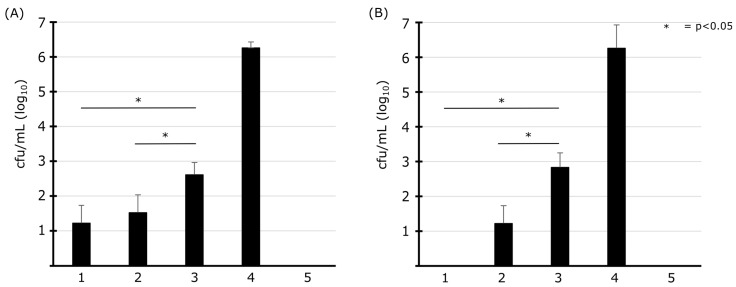
Impact of ClO_2_ depletion and bacterial inactivation: The depletion of active ClO_2_ and the effect on living cells of *E. coli* and *P. aeruginosa* cells were analyzed. Heat-killed *E. coli* (**A**) and *P. aeruginosa* (**B**) cells at 10^3^ and 10^6^ cfu/mL (columns 2 and 3) were mixed with ClO_2_ at a final concentration of 0.9 mg/L for 5 min. Living cells (final concentration 10^6^ cfu/mL) were added for 5 min contact prior to cultivation. The controls consisted of living cells mixed with active (column 1) and inactivated ClO_2_ (column 4). Heat-killed cells (90 °C for 5 min) were taken as negative control (column 5). Statistical comparison of single test approaches based on one-way analysis of variance (ANOVA) and Bonferroni post-test, error bars represent the standard deviations.

Our results give proof of a first fast and efficient microbiological inactivation phase and a subsequent long stagnation phase without a considerable decline of cultivable cells, whereby the effect was recognizable in all microorganisms examined. In addition to the different compositions of highly tolerant and less tolerant subspecies in the bacterial solutions, a loss of the effect of the disinfectant could also play a role. In the following experimental set-up, we investigated the influence of inactivated microorganisms on ClO_2_ regarding a reduction in the efficiency of the disinfectant by the organic burden of microorganisms already present in tap water. Two different concentrations of heat-killed *E. coli* and *P. aeruginosa* cells were mixed with ClO_2_ prior to the application of living cells (Figure 5). Starting from 10^6^ living cfu/mL, the log_10_-levels of reduction amounted to 4.7 and 3.7 for *E. coli* (Figure 5A) and to 5.0 and 3.4 for *P. aeruginosa* (Figure 5B) after pre-incubation with 10^3^ and 10^6^ heat-killed cfu/mL, respectively. Controls without an application of any heat-killed cells resulted in a 5.0 log_10_-levels reduction of *E. coli* cells whereas *P. aeruginosa* was fully eradicated. These results prove a ClO_2_-depletion effect during a short contact time.

## 4. Discussion

Disinfection with ClO_2_ has been proven and evaluated in a variety of applications [30,32,33,34,35,43,44,46,47,48,49]. In this study, we focused on disinfection conditions in an aqueous environment with a high hygienic background level namely DUWLs in dental practices. For this purpose, the effect of ClO_2_ was studied on various relevant bacteria in the planktonic state present in the aqueous environment of DUWLs considering various microbial aspects and factors influencing the effectiveness of ClO_2_ in aqueous solutions.

One approach was to examine the impact of the environmental background solution, in which bacteria exist. Our results indicate that the microbiological background environment had a decisive impact on the successful reduction of bacterial counts. The effect of disinfectants on microorganisms is often investigated in saline and PBS associated with centrifugation steps [45,50]. Since the centrifugation step could be a stress factor for the bacteria leading to cell surface damage and affecting surface-sensitive properties causing significant reductions in viability [51,52], centrifugation was omitted in this study. ClO_2_ has a high bactericidal effect on *E. coli* when present in PBS as it also causes a continuous decrease of CFU over time when suspended in saline. The lowest effect in bacterial reduction was determined for ClO_2_ in tap water. Our findings indicate that either the effect of ClO_2_ in tap water is reduced by naturally occurring organic substances of dead microorganisms or bacteria in aqueous environments have different physiological states. The presence of inorganic substances in tap water has an impact on ClO_2_ depletion, too [53]. As tap water represents the natural background environment of microorganisms existing in pipes and DUWLs, we have chosen tap water as a suspension matrix for bacteria for a better comparison of laboratory conditions and conditions in DUWLs and pipes.

High bacterial contaminations with maximum values between 10^5^–10^6^ cfu/mL were identified in probed DUWLs [1,2]. For this reason, we have analyzed the ClO_2_ efficiency on high cell densities ranging from very high (10^7^ cfu/mL) to quite low (10^4^ cfu/mL). ClO_2_ proved to be highly effective in cell reduction against numerous microorganisms. Regarding our experimental setup, the level of inactivation was higher when supplemented doses were increased. Further, the inactivation effect was also dependent on the density of tested bacteria, which is in contrast to former publications [50,54]. Bacteria in high cell numbers were reduced to a small extent only. The lower the initial cell count, the more cell numbers decreased. This phenomenon was confirmed using various bacteria species. Conspicuously, the main effect of ClO_2_ occurred within the first five minutes of exposure to tested bacteria, which was indicated by low numbers of cultivable cells on agar plates. In the following course of our experiments, the cell counts hardly changed, resulting in a tailing [34,50,54]. This tailing effect at prolonged exposure was observed in various microorganisms studied in this work whereby a ClO_2_ depletion effect should not be disregarded.

Treatment with 1 mg/L ClO_2_ was not sufficient to eradicate *E. coli*, taken from a plate, with a density of 10^5^ cfu/mL in total, which concurs with the result of another study [55]. *P. aeruginosa* from the plate demonstrated this stability as well. However, the *Legionella* species from the plate failed to survive under these conditions, thus after 15 min at the latest, no more growth was observed. These results imply species-related stability or susceptibility to the disinfection procedure. Differences in susceptibility between Gram-negative and Gram-positive microorganisms were observed. *S. aureus* showed higher stability to ClO_2_ treatment than *E. coli.* Our results are consistent with another study, which explained the enhanced stability of the membrane structure and the mechanical stability of *S. aureus* [30]. Gram-negative and Gram-positive bacteria have different membrane structures. Gram-positive bacteria have a thicker peptidoglycan layer than Gram-negative bacteria, which have a double membrane layer [56]. This different susceptibility was evidenced as more than twice the ClO_2_ dose was needed for *S. aureus* to obtain the same log reduction as for *P. aeruginosa* [54].

Microorganisms may change their physiology when living in tap water for an extended time [57]. To simulate the conditions in DUWLs being contaminated with planktonic bacteria, the microorganisms were adapted to tap water and left to survive under a tap water stress situation for several weeks. For *E. coli* and *P. aeruginosa*, the cell count decreased by about 1 log_10_-levels after 11 weeks. Surprisingly, the Gram-positive *S. aureus* was found to survive and be cultivable in tap water over the whole incubation period, although its cell numbers decreased most strongly compared to other species. The long-term survival in tap water allowed a comparison of different physiological conditions between overnight culturing and tap water adaption regarding a change in susceptibility to ClO_2_ treatment. The presence of planktonic cells in tap water for several weeks proves a long-term survival of microorganisms in tap water that might be associated with biofilm formation and therefore, underlines the importance of efficient disinfection of DUWLs. The microbiological stability patterns of ClO_2_ treatment changed when adapted to tap water. High cell numbers were no longer reduced as much as with cultured cells during ClO_2_ treatment but tailing occurred again after a few minutes of exposure. Tap water-adapted cells seemed to be more robust than cultured ones. Adaptation to tap water can cause a change in the cell, such as a less permeable membrane [57] and other physiological changes [58].

As it is shown, ClO_2_ inactivation kinetics corresponded to a two-phase model with a rapid decay in the first minutes followed by plateau formation as described in other studies for bacteria and viruses [38,50,54,59,60]. The occurrence of this tailing phenomenon may be due to several factors at the biological and physical-chemical levels of the disinfectant and microorganisms. Bacteria and viruses in particularly high densities tend to aggregate, resulting in inadequate exposure [50,60]. However, tailing was observed, although samples were intensively vortexed to prevent clumping [50]. One reason for the two-phase model in viruses may be the presence of a ClO_2_-resistant subpopulation [59]. Another study does not exclude the existence of a resistant subpopulation but explains the two-phase model with the change in the properties of the virus during disinfection forming a protective layer on the virus capsid [60]. The presence of a resistant subpopulation could not be excluded as a cause for the two-phase model in bacteria either [50]. Another reason for the appearance of the biphasic kinetics could be a depletion of the disinfectant by organic substances reducing the bactericidal impact of ClO_2_ in solutions [39,48,61]. The pre-load of the ClO_2_ solution with low amounts of heat-killed microorganisms did not show any significant difference for the elimination of *E. coli* and *P. aeruginosa*, but high amounts of heat-killed microorganisms caused a significant reduction of the ClO_2_ effect on the bacterial inactivation level. Even if the effect of ClO_2_ was diminished, an effective part of the disinfectant remained active. The results do not exclude the presence of a ClO_2_ tolerant and stable subpopulation as it was equally reported elsewhere [50,59]. Other reasons, such as the change in the characteristics of the bacteria could also be a reason. In this work, the bacterial density was shown to have a high influence on the sensitivity to ClO_2_, which seems to provide protective measures for bacteria against disinfection. Thus, the tailing effect cannot be attributed to a single factor but it is the result of various interacting aspects. To what extent the change in bacterial properties leads to a tailing effect should be subject to future studies.

## 5. Conclusions

ClO_2_ is a very effective disinfectant against pathogenic and waterborne microorganisms occurring in water systems of dental practices and chair units, where Gram-negative bacteria are more susceptible than Gram-positive. However, in the case of massive contamination with high cell numbers, a substantial number of bacteria can withstand ClO_2_ treatment. When disinfection measures are carried out it should be considered that microorganisms living in tap water might have more robust properties proved by high resistance to ClO_2_ than overnight-cultured bacteria. Furthermore, the environmental background in which the bacteria are suspended might have an impact on the effectiveness of ClO_2_ and the susceptibility of bacteria to the disinfectant. ClO_2_ is an effective disinfectant to waterborne microorganisms in the planktonic state. Further studies aim to investigate conditions for efficient inactivation of sessile and in biofilms living microorganisms in the water systems.

## Figures and Tables

**Table 1:** Microorganisms used in the study.

Strain	No.
*Pseudomonas aeruginosa*	ATCC 27853
*Legionella pneumophila* serogroup 1	ATCC 33152
*Legionella pneumophila* serogroup 5	ATCC 33737
*Legionella anisa*	DSM 17627
*Legionella anisa*	Isolate No. 137
*Escherichia coli*	ATCC 25922
*Staphylococcus aureus*	ATCC 6538

## Data Availability

The authors declare that all data obtained have been included in the manuscript.

## References

[1] Abel L.C., Miller R.L., Micik R.E., Ryge G. (1971). Studies on Dental Aerobiology: IV. Bacterial Contamination of Water Delivered by Dental Units. J. Dent. Res..

[2] Forde A., O’Reilly P., Fitzgerald G., O’Mullane D., Burke F.M., O’Sullivan M. (2005). Microbial contamination of dental unit water systems. J. Ir. Dent. Assoc..

[3] Alkhulaifi M.M., Alotaibi D.H., Alajlan H., Binshoail T. (2020). Assessment of nosocomial bacterial contamination in dental unit waterlines: Impact of flushing. Saudi Dent. J..

[4] Lipphaus P., Hammes F., Kötzsch S., Green J., Gillespie S., Nocker A. (2013). Microbiological tap water profile of a medium-sized building and effect of water stagnation. Environ. Technol..

[5] Lautenschlager K., Boon N., Wang Y., Egli T., Hammes F. (2010). Overnight stagnation of drinking water in household taps induces microbial growth and changes in community composition. Water Res..

[6] Bagga B.S.R., Murphy R.A., Anderson A.W., Punwani I. (1984). Contamination of dental unit cooling water with oral microorganisms and its prevention. J. Am. Dent. Assoc..

[7] Lewis D.L., Boe R.K. (1992). Cross-infection risks associated with current procedures for using high-speed dental handpieces. J. Clin. Microbiol..

[8] Whitehouse R., Peters E., Lizotte J., Lilge C. (1991). Influence of biofilms on microbial contamination in dental unit water. J. Dent..

[9] Micik R.E., Miller R.L., Mazzarella M.A., Ryge G. (1969). Studies on Dental Aerobiology: I. Bacterial Aerosols Generated during Dental Procedures. J. Dent. Res..

[10] Boccia G., Di Spirito F., D’ambrosio F., De Caro F., Pecora D., Giorgio R., Fortino L., Longanella W., Franci G., Santella B. (2023). Microbial Air Contamination in a Dental Setting Environment and Ultrasonic Scaling in Periodontally Healthy Subjects: An Observational Study. Int. J. Environ. Res. Public Health.

[11] Infektionsprävention in der Zahnheilkunde-Anforderungen an die Hygiene (2006). Mitteilung der Kommission für Krankenhaushygiene und Infektionsprävention beim Robert Koch-Institut. Bundesgesundheitsbl Gesundh. Gesundh..

[12] Lück P.C., Leupold I., Hlawitschka M., Helbig J.H., Carmienke I., Jatzwauk L., Guderitz T. (1993). Prevalence of Legionella species, serogroups, and monoclonal subgroups in hot water systems in south-eastern Germany. Zent. Hyg. Umweltmed..

[13] Atlas R.M., Williams J.F., Huntington M.K. (1995). Legionella contamination of dental-unit waters. Appl. Environ. Microbiol..

[14] Fleres G., Couto N., Lokate M., Van der Sluis L.W.M., Ginevra C., Jarraud S., Deurenberg R.H., Rossen J.W., García-Cobos S., Friedrich A.W. (2018). Detection of *Legionella anisa* in Water from Hospital Dental Chair Units and Molecular Characterization by Whole-Genome Sequencing. Microorganisms.

[15] Arnow P.M., Chou T., Weil D., Shapiro E.N., Kretzschmar C. (1982). Nosocomial Legionnaires’ Disease Caused by Aerosolized Tap Water from Respiratory Devices. J. Infect. Dis..

[16] Cordes L.G., Fraser D.W. (1980). Legionellosis: Legionnaires’ disease; Pontiac fever. Med. Clin. N. Am..

[17] Rousseau C., Ginevra C., Simac L., Fiard N., Vilhes K., Ranc A.-G., Jarraud S., Gornes H., Mouly D., Campese C. (2022). A Community Outbreak of Legionnaires’ Disease with Two Strains of *L. pneumophila* Serogroup 1 Linked to an Aquatic Therapy Centre. Int. J. Environ. Res. Public Health.

[18] Ricci M.L., Fontana S., Pinci F., Fiumana E., Pedna M.F., Farolfi P., Sabattini M.A.B., Scaturro M. (2012). Pneumonia associated with a dental unit waterline. Lancet.

[19] Schönning C., Jernberg C., Klingenberg D., Andersson S., Pääjärvi A., Alm E., Tano E., Lytsy B. (2017). Legionellosis acquired through a dental unit: A case study. J. Hosp. Infect..

[20] Lyczak J.B., Cannon C.L., Pier G.B. (2000). Establishment of Pseudomonas aeruginosa infection: Lessons from a versatile opportunist. Microbes Infect..

[21] Mena K.D., Gerba C.P. (2009). Risk Assessment of Pseudomonas aeruginosa in Water. Rev. Environ. Contam. Toxicol..

[22] Fitzgibbon E.J., Bartzokas C.A., Martin M.V., Gibson M.F., Graham R. (1984). The source, frequency and extent of bacterial contamination of dental unit water systems. Br. Dent. J..

[23] Tesauro M., Consonni M., Grappasonni I., Lodi G., Mattina R. (2022). Dental unit water content and antibiotic resistance of *Pseudomonas aeruginosa* and *Pseudomonas* species: A case study. J. Oral Microbiol..

[24] Martin M.V. (1987). The significance of the bacterial contamination of dental unit water systems. Br. Dent. J..

[25] Lowy F.D. (1998). Staphylococcus aureus Infections. N. Engl. J. Med..

[26] Kellett M., Holbrook W. (1980). Bacterial contamination of dental handpieces. J. Dent..

[27] Tuvo B., Totaro M., Cristina M.L., Spagnolo A.M., Di Cave D., Profeti S., Baggiani A., Privitera G., Casini B. (2020). Prevention and Control of *Legionella* and *Pseudomonas* spp. Colonization in Dental Units. Pathogens.

[28] Karpay R.I., Plamondon T.J., Mills S.E., Dove S.B. (1999). Combining periodic and continuous sodium hypochlorite treatment to control biofilms in dental unit water systems. J. Am. Dent. Assoc..

[29] Martiny H., Kampf W.-D., Rüden H., Gundermann K.-O., Rüden H., Sonntag H.-G. (1991). Antimikrobielle Verfahren und Entwesung: Desinfektion. Grundlagen. Lehrbuch der Hygiene: Umwelthygiene, Krankenhaushygiene, Individualhygiene, Sozialhygiene und öffentliches Gesundheitswesen, Epidemiologie.

[30] Huang J., Wang L., Ren N., Ma F., Juli (1997). Disinfection effect of chlorine dioxide on bacteria in water. Water Res..

[31] Shen C., Norris P., Williams O., Hagan S., Li K. (2016). Generation of chlorine by-products in simulated wash water. Food Chem..

[32] Bansal R., Puttaiah R., Harris R., Reddy A. (2011). Evaluation of Two Methods in Controlling Dental Treatment Water Contamination. J. Contemp. Dent. Pract..

[33] Wei L.-L., Hu C.-C., Hsu C.-W., Pen C.-W., Chen L.-Y., Yu Y.-C., Carey J.R., Yin H.-C., Wang S.-S. (2021). Disinfection of Dental Chair Water Using Aqueous Chlorine Dioxide. Water.

[34] Benarde M.A., Israel B.M., Olivieri V.P., Granstrom M.L. (1965). Efficiency of Chlorine Dioxide as a Bactericide. Appl. Microbiol..

[35] Gordon G., Rosenblatt A.A. (2005). Chlorine Dioxide: The Current State of the Art. Ozone Sci. Eng..

[36] Yang X., Guo W., Lee W. (2013). Formation of disinfection byproducts upon chlorine dioxide preoxidation followed by chlorination or chloramination of natural organic matter. Chemosphere.

[37] Wen G., Xu X., Huang T., Zhu H., Ma J. (2017). Inactivation of three genera of dominant fungal spores in groundwater using chlorine dioxide: Effectiveness, influencing factors, and mechanisms. Water Res..

[38] Jin M., Shan J., Chen Z., Guo X., Shen Z., Qiu Z., Xue B., Wang Y., Zhu D., Wang X. (2013). Chlorine Dioxide Inactivation of Enterovirus 71 in Water and Its Impact on Genomic Targets. Environ. Sci. Technol..

[39] Hassenberg K., Praeger U., Herppich W.B. (2021). Effect of Chlorine Dioxide Treatment on Human Pathogens on Iceberg Lettuce. Foods.

[40] Hinenoya A., Awasthi S.P., Yasuda N., Shima A., Morino H., Koizumi T., Fukuda T., Miura T., Shibata T., Yamasaki S. (2015). Chlorine Dioxide is a Better Disinfectant than Sodium Hypochlorite against Multi-Drug Resistant *Staphylococcus aureus*, *Pseudomonas aeruginosa*, and *Acinetobacter baumannii*. Jpn. J. Infect. Dis..

[41] Moody L.V., Miyamoto Y., Ang J., Richter P.J., Eckmann L. (2019). Evaluation of Peroxides and Chlorine Oxides as Disinfectants for Chemical Sterilization of Gnotobiotic Rodent Isolators. J. Am. Assoc. Lab. Anim. Sci..

[42] Rosende M., Miró M., Salinas A., Palerm A., Laso E., Frau J., Puig J., Matas J.M., Doménech-Sánchez A. (2020). Cost-Effectiveness Analysis of Chlorine-Based and Alternative Disinfection Systems for Pool Waters. J. Environ. Eng..

[43] Sorlini S., Gialdini F., Biasibetti M., Collivignarelli C. (2014). Influence of drinking water treatments on chlorine dioxide consumption and chlorite/chlorate formation. Water Res..

[44] Van Haute S., Tryland I., Escudero C., Vanneste M., Sampers I. (2017). Chlorine dioxide as water disinfectant during fresh-cut iceberg lettuce washing: Disinfectant demand, disinfection efficiency, and chlorite formation. LWT.

[45] Isomoto H., Urata M., Kawazoe K., Matsuda J., Nishi Y., Wada A., Ohnita K., Hirakata Y., Matsuo N., Inoue K. (2006). Endoscope disinfection using chlorine dioxide in an automated washer-disinfector. J. Hosp. Infect..

[46] Vincenti S., de Waure C., Raponi M., Teleman A.A., Boninti F., Bruno S., Boccia S., Damiani G., Laurenti P. (2018). Environmental surveillance of Legionella spp. colonization in the water system of a large academic hospital: Analysis of the four–year results on the effectiveness of the chlorine dioxide disinfection method. Sci. Total. Environ..

[47] Dallolio L., Scuderi A., Rini M.S., Valente S., Farruggia P., Sabattini M.A.B., Pasquinelli G., Acacci A., Roncarati G., Leoni E. (2014). Effect of Different Disinfection Protocols on Microbial and Biofilm Contamination of Dental Unit Waterlines in Community Dental Practices. Int. J. Environ. Res. Public Health.

[48] Chang C.-Y., Hsieh Y.-H., Hsu S.-S., Hu P.-Y., Wang K.-H. (2000). The formation of disinfection by-products in water treated with chlorine dioxide. J. Hazard. Mater..

[49] Hamilton E., Seal D., Hay J. (1996). Comparison of chlorine and chlorine dioxide disinfection for control of Legionella in a hospital potable water supply. J. Hosp. Infect..

[50] Ofori I., Maddila S., Lin J., Jonnalagadda S.B. (2017). Chlorine dioxide oxidation of *Escherichia coli* in water—A study of the disinfection kinetics and mechanism. J. Environ. Sci. Health Part A.

[51] Peterson B.W., Sharma P.K., van der Mei H.C., Busscher H.J. (2012). Bacterial Cell Surface Damage Due to Centrifugal Compaction. Appl. Environ. Microbiol..

[52] Liu Z., Carroll Z.S., Long S.C., Roa-Espinosa A., Runge T. (2017). Centrifuge separation effect on bacterial indicator reduction in dairy manure. J. Environ. Manag..

[53] Gan W., Ge Y., Zhong Y., Yang X. (2020). The reactions of chlorine dioxide with inorganic and organic compounds in water treatment: Kinetics and mechanisms. Environ. Sci. Water Res. Technol..

[54] Ofori I., Maddila S., Lin J., Jonnalagadda S.B. (2018). Chlorine dioxide inactivation of Pseudomonas aeruginosa and Staphylococcus aureus in water: The kinetics and mechanism. J. Water Process. Eng..

[55] Hassenberg K., Geyer M., Mauerer M., Praeger U., Herppich W.B. (2017). Influence of temperature and organic matter load on chlorine dioxide efficacy on Escherichia coli inactivation. LWT Food Sci. Technol..

[56] Silhavy T.J., Kahne D., Walker S. (2010). The Bacterial Cell Envelope. Cold Spring Harb. Perspect. Biol..

[57] Lewenza S., Abboud J., Poon K., Kobryn M., Humplik I., Bell J.R., Mardan L., Reckseidler-Zenteno S. (2018). Pseudomonas aeruginosa displays a dormancy phenotype during long-term survival in water. PLoS ONE.

[58] Mendis N., Lin Y.R., Faucher S.P. (2014). Comparison of virulence properties of Pseudomonas aeruginosa exposed to water and grown in rich broth. Can. J. Microbiol..

[59] Hornstra L., Smeets P., Medema G. (2011). Inactivation of bacteriophage MS2 upon exposure to very low concentrations of chlorine dioxide. Water Res..

[60] Sigstam T., Rohatschek A., Zhong Q., Brennecke M., Kohn T. (2014). On the cause of the tailing phenomenon during virus disinfection by chlorine dioxide. Water Res..

[61] Ayyildiz O., Ileri B., Sanik S. (2009). Impacts of water organic load on chlorine dioxide disinfection efficacy. J. Hazard. Mater..

